# Electrical Stimulation Exercise for People with Spinal Cord Injury: A Healthcare Provider Perspective

**DOI:** 10.3390/jcm12093150

**Published:** 2023-04-27

**Authors:** David R. Dolbow, Ashraf S. Gorgey, Therese E. Johnston, Ines Bersch

**Affiliations:** 1 Department of Physical Therapy, College of Osteopathic Medicine, William Carey University, Hattiesburg, MS 39401, USA; 2 Spinal Cord Injury and Disorders Center, Hunter Holmes McGuire VA Medical Center, Richmond, VA 23249, USA; 3 College of Medicine, Virginia Commonwealth University, Richmond, VA 23298, USA; 4 Department of Physical Therapy, Arcadia University, Glenside, PA 19038, USA; 5 International FES Centre^®^, Swiss Paraplegic Center, CH-6207 Nottwil, Switzerland

**Keywords:** peripheral nerve stimulation, functional electrical stimulation, neuromuscular electrical stimulation, spinal cord injury

## Abstract

Electrical stimulation exercise has become an important modality to help improve the mobility and health of individuals with spinal cord injury (SCI). Electrical stimulation is used to stimulate peripheral nerves in the extremities to assist with muscle strengthening or functional activities such as cycling, rowing, and walking. Electrical stimulation of the peripheral nerves in the upper extremities has become a valuable tool for predicting the risk of hand deformities and rehabilitating functional grasping activities. The purpose of this paper is to provide healthcare providers perspective regarding the many rehabilitation uses of electrical stimulation in diagnosing and treating individuals with SCI. Electrical stimulation has been shown to improve functional mobility and overall health, decrease spasticity, decrease the risk of cardiometabolic conditions associated with inactivity, and assist in the diagnosis/prognosis of hand deformities in those with tetraplegia. Studies involving non-invasive stimulation of the spinal nerves via external electrodes aligned with the spinal cord and more invasive stimulation of electrodes implanted in the epidural lining of the spinal cord have demonstrated improvements in the ability to stand and enhanced the stepping pattern during ambulation. Evidence is also available to educate healthcare professionals in using functional electrical stimulation to reduce muscle spasticity and to recognize limitations and barriers to exercise compliance in those with SCI. Further investigation is required to optimize the dose-response relationship between electrical stimulation activities and the mobility and healthcare goals of those with SCI and their healthcare providers.

## 1. Introduction

Spinal cord injury (SCI) disrupts efferent and afferent pathways, including the descending pathways from the motor cortex to the spinal motor neurons which activate muscle activity [1]. Electrical stimulation can be used to bypass spinal disruption and elicit muscle contractions for rehabilitation purposes [1]. Electrical stimulation exercise has become an important modality for improving the health and mobility of individuals with SCI [2,3]. The two primary types of electrical stimulation producing exercise are (1) peripheral nerve stimulation (PNS), which is the electrical stimulation of the peripheral nerves, usually in the extremities, and (2) spinal cord stimulation (SCS), which is the electrical stimulation of the spinal nerves at the spinal cord [4]. PNS is further classified into neuromuscular electrical stimulation (NMES) and functional electrical stimulation (FES). While some people use the two terms interchangeably, the two modalities are commonly separated into two categories. NMES is defined as electrically induced muscle contractions, which includes resistance training [5]. FES is defined as electrically induced functional activities, including FES cycling, FES rowing, FES walking, and FES-assisted grasping activities [6,7,8,9]. Similarly, SCS is further divided into transcutaneous spinal stimulation and epidural electrical stimulation [3,9,10]. Transcutaneous spinal stimulation provides electrical input via surface electrodes aligned along the external surface of the spine, while epidural stimulation is derived from electrodes surgically implanted in the epidural spaces of the spinal cord [10,11,12]. Three primary electrical stimulation parameters are adjusted to optimize the activity of interest: pulse duration (time duration for a single pulse), frequency (pulses produced per second), and amplitude (strength of the current) that is often referred to as stimulation intensity [13].

The potential benefits of electrical stimulation in individuals with SCI may include changes in body composition, such as increasing muscle mass and bone mass while decreasing fat mass; improving cardiovascular and metabolism efficiency; decreasing spasticity; and improving functional mobility [2,8,14,15,16]. In addition, electrical stimulation can be used as a diagnostic tool for determining lower motor neuron damage caused by cervical SCI which may affect the risk of developing grasping anomalies [16]. Most recently, electrical stimulation has been employed to guide clinicians and researchers in the estimation of the quantities of muscle and bone. During NMES, Gorgey and colleagues used the amplitude of the current (<100 mA) and the number of leg extension repetitions (>70) as cut-offs to provide both diagnostic and prognostic assessments of the muscle cross-sectional area and knee bone mineral densities in persons with SCI [17].

Electrical stimulation activities have been shown to be safe with proper supervision and instruction by healthcare professionals. Telehealth monitoring has also been successfully used for the application of home-based NMES. Participants were educated to use surface NMES to induce resistance training exercises of the knee extensors and were monitored over an eight-week period via telehealth [18]. However, some important contraindications and precautions should be recognized, including very low bone density; a history of bone fractures; uncontrolled autonomic dysreflexia; uncontrolled hyper/hypotension; open pressure wounds; thrombosis; pregnancy; cancer; pacemaker and defibrillator, depending on the on distance from the implant; and orthopedic problems that preclude the selected activity [19]. The purpose of this paper is to provide a healthcare provider’s perspective regarding the many rehabilitation uses of electrical stimulation in diagnosing and treating individuals with SCI.

## 2. Body Composition Assessment

Along with optimizing functional mobility, healthcare providers use FES activities to decrease the risk of secondary conditions after SCI, including cardiometabolic diseases which is two to three times greater than in the able-bodied population [20]. Two recent systematic literature reviews concluded that NMES and FES activities provided positive results on muscle mass after eight to sixteen weeks of training [21,22]. Atkins and Bickel [21] reported increases in muscle volume that range from 20–72% with an average increase of 26% among NMES and FES studies. Similarly, Bekhet et al. [22] reported skeletal muscle increases in cross-section areas (CSA) from 5.7% to 75% via NMES and FES, with NMES resistance training typically providing greater increases in muscle than FES cycling. 

**Figure 1:**
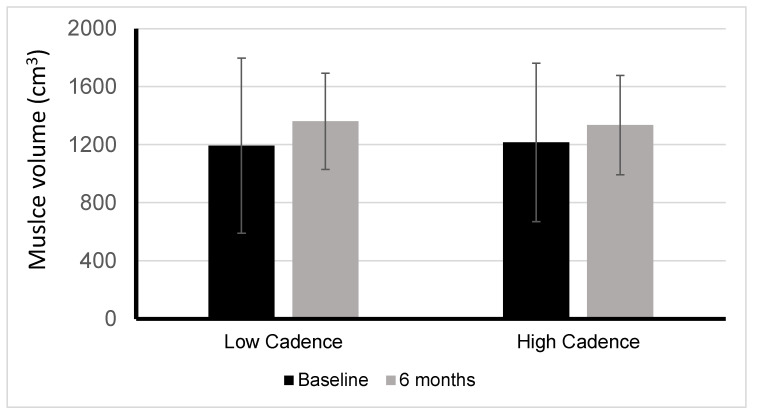
Muscle volume changes between low versus high cadence FES-LEC. Changes in muscle volume after six months of low cadence/high torque cycling (LOW) or high cadence/low torque cycling (HIGH). Between groups *p* = 0.318; within groups LOW *p* = 0.014 and HIGH *p* = 0.049.

Johnston et al. [6] demonstrated that there is a significant correlation between accumulated torque and muscle volume. In a comparison between low cadence/high torque and high cadence/low torque FES cycling groups, 17 adults with chronic C4-T6 motor complete SCI all increased in muscle volume, but the low cadence/high torque group had a 9% greater increase (Figure 1). Participants cycled three times per week for six months. In a study of ten adults with chronic SCI, Dolbow et al. [23] found a 5.7% increase in lean leg mass and a 2.4% decrease in body fat percentage when combining resistance-guided high-intensity interval FES cycling three times per week for eight weeks with once weekly nutritional counseling. 

A nutritional counseling-only group displayed no changes in body composition. Similarly, Gorgey et al. [24] compared NMES resistance training plus nutritional counseling twice a week for twelve weeks to a control group that received nutritional counseling only. The results showed skeletal muscle CSA increases of 28% for the whole thigh, 35% for the knee extensors, and 16% for the knee flexors for those that received NMES and nutritional counseling. Additionally, there was a 25% increase in insulin-like growth factor 1 (IGF-1) which is associated with muscle hypertrophy. Interestingly, there was a concomitant 25% reduction in visceral adipose tissue (VAT) CSA in the L5-S3 region. Gorgey and Shepard [25] published a unilateral leg case report using NMES resistance training twice a week for twelve weeks on an individual with chronic cervical SCI. The results revealed a 72% increase in CSA of the whole thigh and a 53% decrease in intramuscular fat. This initial report was later confirmed in a randomized clinical trial that demonstrated the efficacy of utilizing NMES-RT with and without androgen replacement therapy to evoke muscle hypertrophy and provide other favorable outcomes similar to an increase in basal metabolic rate and enhancing carbohydrate profile in persons with SCI [24,26]. 

Ye et al. [27] completed a systematic review of the literature on FES rowing exercises on individuals with SCI and reported mixed results in body composition. For example, Kim et al. [28] found a 14% decrease in body fat percentage and a 5.8% increase in muscle mass after six weeks of training, while Jeon et al. [29] and Wilbanks et al. [30] found no change in body fat after FES rowing training after twelve and six weeks respectively. A recent randomized clinical trial demonstrated that, compared to passive movement training, NMES-RT induced 30% muscle hypertrophy accompanied by a 14% increase in oxygen uptake. The authors also reported improvement in indices of cardiovascular performance as measured by ventilation/carbon dioxide production (VE/VCO_2_) [31]. Interestingly, the same study noted an increase in whole body fat utilization as a primary source of energy expenditure during FES lower extremities cycling at low intensity. The study may conceivably shed light on the significance of evoking muscle hypertrophy prior to enhancing the cardio-metabolic profile with FES lower extremities cycling [32]. 

Due to the slower metabolic processes in bone compared to muscle and fat, it is widely accepted that interventions require several months or more to produce significant changes in bone mass [33]. Positive results on bone mineral density have been sparse and relatively inconsistent when examining the most fracture-prone sites (distal and proximal femur and tibia) in those with SCI [34,35]. The Clinical Practice Guidelines provided by Craven et al. [36] suggested FES cycling, FES rowing, or NMES resistance exercise as possible options for preventing the decline of bone mineral density in the hip and knee regions for individuals with SCI. They further recommend a pulse duration of ≥200 µs, 20–33 Hz for frequency, and an amplitude/intensity of up to 140 mA that should create strong visible muscle contractions. For exercise duration, they recommend three to five days per week for thirty or more minutes per session for at least a year to see changes in bone. 

## 3. Cardiovascular and Metabolism

In a systematic review of the literature, van der Scheer et al. [37] found that 16 out of 21 selected studies using electrical stimulation exercise for individuals with SCI showed improvements in cardiovascular and metabolic outcomes. The most common outcomes measured were power output and peak oxygen volume (VO2peak). These studies provide consistent evidence that FES cycling can improve aerobic fitness and has the potential to reduce the risk of cardiovascular and metabolic conditions after SCI. On the contrary, Hamzaid and Davis [38] concluded that the lack of consistency in the various studies resulted in insufficient evidence to determine if health and fitness benefits could be derived from FES exercise in individuals with SCI. They suggested that the VO2peak utilized may be more limited due to the reduced active muscle mass and peripheral blood flow than due to central cardiac reserve. Figoni and Dolbow [39] studied the possible benefits of aerobic exercise on those with tetraplegia and concluded that, while further study is needed, the current evidence suggests that the greatest cardiovascular and metabolic benefits derived from FES cycling are likely to result from thirty or more minutes of moderate-intensity exercise, three or more times per week for at least eight to twelve weeks. Studies investigating training programs using FES cycling and FES rowing for individuals with SCI have reported improved VO2peak, lowered blood glucose levels, and enhanced skeletal muscle glucose uptake [27,29,40]. Akins and Bickel [21] summarized the effects of FES activities on metabolic health by suggesting that FES interventions can help to normalize glucose uptake and metabolism after SCI. FES activities also have been shown to improve energy expenditure, increase cardiac output, reverse myocardial atrophy, increase cardiac protective high-density lipoprotein cholesterol, and assist in the reduction of body fat percentage [40,41,42,43].

## 4. Muscle Spasticity

Muscle spasticity is a common condition secondary to SCI and can potentially increase the level of disability [44]. Spasticity typically results from upper motor neuron injury in those with injuries above T12/L1 [44]. The SCI reduces or eliminates control of reflexes from the supraspinal level of the central nervous system resulting in spasticity that is characterized by increased muscle tone, hyperreflexia, clonus sign, and muscle spasms [44]. NMES is thought to improve spasticity by eliciting disynaptic reciprocal inhibition of the opposing muscle group Alashram et al. [44] completed a systematic review investigating the changes in lower extremities’ spasticity after FES cycling. The investigation included ten independent studies totaling 161 individuals with SCI. Alashram and associates concluded that more randomized control trials are needed; however, current evidence indicates that FES cycling can reduce lower extremities’ spasticity for individuals at all levels of SCI. It is interesting to note that ankle dorsiflexor and plantarflexor spasticity have been shown to be reduced during FES cycling even though electrical stimulation is provided to the quadriceps, hamstrings, and gluteal muscles [44]. 

Fang et al. [45] completed a twelve-study systematic review of the qualitative data involving spasticity after SCI and an eight-study meta-analysis on quantitative data regarding spasticity after SCI. The eight studies used in the meta-analysis included a total of 99 participants with SCI. Fang and associates concluded that FES cycling can decrease spasticity for individuals with SCI. Other important evidence highlighted by Fang and colleagues concerning the use of FES cycling to decrease spasticity include that (1) the correlation between the number of FES cycling sessions and the level of decrease in spasticity is not linear; and (2) generally, about twenty FES cycle training sessions need to be completed to obtain the efficacy to decrease spasticity. More specifically, six studies reported by Fang and colleagues used multiple FES cycling sessions as the intervention and found that the post-intervention Modified Ashworth Scores (MAS) for the lower extremities were significantly decreased from the pre-intervention scores. Two other studies reported by the same authors found decreased MAS after a single bout of FES cycling. 

## 5. Exercise Adherence

A national health survey by the Centers for Disease Control and Prevention in 2020 [46] determined that only 24.2% of adults aged 18 and over met the physical activity guidelines for Americans for both aerobic and muscle-strengthening activities (150 min per week of moderate-intensity aerobic exercise and muscle strengthening exercises to the major muscle groups twice per week) [46]. Individuals with SCI perform only 35–40% as much exercise as the largely sedentary able-bodied population, demonstrating the extreme lack of physical activity in the SCI population [47,48]. Recently, Tui et al. [49] completed a qualitative study concerning the motivations and barriers that limit adherence to exercise programs for individuals with SCI. The common self-reported reasons for poor adherence to exercise guidelines for those with SCI were time constraints (54%), lack of motivation (31%), decreased accessibility (24%), and SCI-specific barriers (23%). The 144 participants in the study reported the possible following solutions: scheduling exercise sessions for time constraints (47.9%); introducing fun during the exercise sessions to increase motivation (21.8%); providing equipment to allow home exercise (30.3%); and locating accessible facilities to resolve accessibility barriers (27.3%). In agreement that access to exercise facilities is a problem, Dolbow and Figoni [50] investigated the accommodation of wheelchair users by community fitness centers and found accommodation lacking, especially regarding access to exercise equipment. 

Dolbow et al. [48] investigated exercise adherence in a home-based FES cycling program for 17 chronic SCI adults for two consecutive eight-week exercise periods. Participation during the first eight weeks was incentivized with the knowledge that the rented FES cycle would be purchased for the participants if they maintained good exercise adherence with the requested 30 to 40 min FES cycling sessions three times per week for the eight weeks. The second eight weeks of FES cycling provided no incentive for participation. During the first eight weeks, the adherence rate to the exercise program was 71.7% while, during the second eight weeks, exercise adherence was 63.7%, a nominal but not statistically significant decrease. The main factors involved with higher adherence rates were age (under 50 years of age had a higher adherence rate); self-reported prior history of regular exercise; and having a history of recurrent pain but finding the FES cycling activity to be pain-free. The last factor fostered the development of the Pain-Free Affinity Model which states that “when living with frequent or recurrent pain, there is an increased affinity toward activities that are perceived as pain-free” [48]. The level of injury, time since injury, and history of depression did not significantly affect exercise adherence in the study. Another follow-up study determined the feasibility of a video conferencing approach as a telehealth communication to deliver a home-based NMES-RT program for eight weeks. The authors intentionally performed unilateral training on one leg while the other leg served as the control. The training paradigm was successful in enhancing muscle hypertrophy in the trained leg but not in the control limb [18]. The telehealth paradigms were important because, even with a short period of de-training or dose de-escalation, persons with SCI experience a gradual loss in muscle size and a decline in cardio-metabolic gains after a routine training program [51]. Today, the telehealth home-based training paradigm via video conferencing has been extended to implement 12 months of training for persons with lower motor neuron injury [52]. 

## 6. Physical Function

Sadowsky et al. [53] hypothesized that restoring normal activity levels should optimize neural regeneration after SCI. This hypothesis was supported by their retrospective cohort cross-sectional evaluation comparing twenty-five people with chronic SCI who underwent an activity-based restorative exercise program including FES cycling to twenty individuals with SCI that received regular standard of care therapy, including a range of motion exercises and stretching of the paralyzed limbs. The participants were matched by age, gender, injury level, the severity of the injury, and duration of the injury. After 29 months, those in the FES cohort demonstrated an 80% increase in neurological function, including motor and sensory advancements as shown on the American Spinal Injury Association Impairment scale. This was a more statistically significant increase than the 40% increase shown by the group receiving the regular standard of care. 

The systematic review and meta-analysis performed by Fang et al. [45] included two studies that measured functional walking gains induced by FES cycling in individuals with SCI via the Six Minute Walk Test (6MWT) and the Timed Get Up and Go Test (TUG). The evidence displayed significantly improved scores in the 6MWT and TUG after FES cycling [54,55]. In those two studies, Kuhn et al. [54] investigated the effects of FES cycling for 20-min sessions twice a week for four weeks. In addition, Mazzoleni and colleagues [55] combined 20 sessions of FES cycling followed by 20 training sessions of exoskeleton overground walking. 

Transcutaneous spinal cord electrical stimulation can be considered a form of FES walking although the activity is unique due to the noninvasive stimulation of the nerves along the lumbosacral region of the spine. This relatively recent innovation in electrical stimulation therapy allows neuromodulation of the spinal circuitry promoting an effective stepping motion that may potentially fine-tune locomotion for those with SCI [56]. Different leg muscles can be stressed by altering the placement of electrodes along the spine, depending on the needs of the individual. For example, low transcutaneous spinal-cord stimulation intensities at the T10–T11 segment produced a higher magnitude response in the vastus lateralis and rectus femoris with a lesser magnitude in the medial gastrocnemius, soleus, and medial hamstrings muscles. The same intensity stimulation at the T12-L1 segment created the reverse relationship of these muscle groups [56].

Sutor et al. [10] combined the use of transcutaneous spinal cord electrical stimulation with the use of a robotic exoskeleton system. Exoskeleton walking uses an external application to provide passive/assistive locomotion involving the legs. However, when it was combined with the transcutaneous spinal cord electrical stimulation over the lumbosacral area of the spine, participants were able to produce significantly more steps and display greater quadricep muscle electromyographic activity than those using the exoskeleton alone. Transcutaneous spinal cord stimulation is considered noninvasive with the external placement of the cathode over the posterior of the spine and the anodes placed bilaterally over the iliac crests [10]. The applications of transcutaneous spinal stimulation (TSS) have extended to include enhancement of upper extremity functions in persons with SCI, spasticity control, and bladder functions. Gad et al. [9] noted that just four weeks of TSS resulted in improvement in unilateral and bilateral hand dexterity. Many daily life activities have been significantly improved following TSS applications on the cervical neural circuitries. Research showed that TSS applications may be associated with cortical inhibition as well as an increased level of excitability of spared dormant spinal axonal tracts at the level of injury. A recent scoping review has included a summary of the research studies and protocols that implemented different TSS techniques to enhance upper and lower extremity motor functions in persons with SCI [57]. The scoping review highlighted that there is inconclusive evidence concerning the exact neurophysiological mechanism by which TSS augments motor functions after SCI. This mechanism may include cortical inhibition, direct stimulation of the dorsal nerve roots, or activation of the spared spinal cord circuitry. It is also unclear whether the amplitude of the current should be set at the motor threshold level, sub-motor, or supra-motor threshold levels. The authors also attempted to summarize the impact of different anatomical placements of the cathodal electrodes, single versus multiple sites, and different waveforms. Finally, the influence of carrier frequency on the neuromodulation capacity of TSS was raised with some studies favoring the application of the carrier frequency (5–10 kHz) and other studies not supporting its applications. The rationale of using carrier frequencies is based on using interferential current that may facilitate deep penetration to stimulate the neural circuitries and reduce painful feelings or sensations underneath the stimulating electrodes. 

Today, our experience in applications of TSS is rudimentary and based on a limited number of randomized clinical trials and published case reports. Others have utilized TSS in conjunction with exoskeleton training and noted enhancement in motor performance, [58,59] reduction in spasticity, and an improvement in autonomic profile in persons with SCI. Currently, there is limited evidence that non-invasive TSS may be a feasible rehabilitation strategy that can facilitate motor control to restore locomotion in persons with SCI [60,61]. 

Spinal cord epidural stimulation (SCES) is an experimental approach that can be either surgically implanted using a paddle or percutaneous leads to enhance motor function, autonomic regulation, and bladder function, and to reduce spasticity. Most of these case reports and case series showed beneficial effects on restoring motor function as demonstrated by enhancing overground ambulation with a walking aid [62,63,64,65,66,67]. The use of percutaneous leads for SCES has been recommended for a century in the reduction of spasticity and restoration of motor control around specific joints. Whether the less invasive percutaneous SCES has the potential to restore motor function similar to the paddle implantation is yet to be determined. 

It is still difficult to indicate whether participants were able to restore total functional ambulation on a daily basis or become less dependent on their wheelchairs based on the published reports. Today, the exact mechanisms of how SCES may influence motor control to enhance functional recovery have yet to be explored [68]. Shah and Lavrov [68] investigated optimal stimulation configurations for neuromodulation of the stepping pattern in spinally transected rats. They found that the stepping patterns were better during a stimulation frequency of 30–40 Hz at the second lumbar and first sacral segments compared to lower frequencies of 5–20 Hz. Stimulation frequency of greater than 50 Hz produced poorer stepping patterns. Improved stepping patterns were found after six training sessions as early as three weeks post-injury. Harkema et al. [68] demonstrated that using SCES below the level of injury (lumbosacral region) was helpful in improving the standing and stepping ability of a 23-year-old male three to four years after a motor vehicle accident causing C7T1 paraplegia. 

Gill et al. [12] used task-specific training with implanted SCES to produce bilateral stepping on a treadmill, independent from trainer assistance or body-weight support in an individual with complete T6 paraplegia. Walking speed increased from 0.05 to 0.20 m per second. The restoration of ambulation required over 100 SCES sessions for 43 weeks. In addition to the treadmill training, the individual also underwent independent stepping overground ambulation with a front-wheeled walker and trainer assistance with balance at the hips. Gorgey et al. [69] also found improved stepping with the combination of SCES with exoskeletal-assisted walking in a case report involving a participant with complete C7 SCI. After 24 sessions of exoskeletal-assisted walking with SCES, volitional stepping was achieved with a reduction of the swing phase assistance from the exoskeletal system from 100–35%. Temporal and rhythmic improvements were also captured with electromyography (EMG) of muscle patterns in the lower extremities. 

While both transcutaneous spinal and epidural electrical stimulation provide potential rehabilitation benefits including increased muscle activity and the facilitation of standing and overground ambulation [3], rodent participants have shown greater improvements than human participants with SCES of the spine. One possible reason for this difference in results is that, in humans, there may be more interference between electrical stimulation and proprioceptive information.

Formento et al. [70] hypothesized that this interference prevents the modulation of the reciprocal inhibitory system used during walking and decreases leg position awareness. However, there is evidence that proprioceptive information can be preserved through alteration in the spaciotemporal stimulation protocols [70]. Angeli et al. [66] successfully demonstrated the benefits of customizing SCES on two individuals with chronic post-traumatic SCI. The electrical stimulation was combined with stepping on a treadmill, over-ground standing, and over-ground walking. The electrical stimulation configurations were modified every two to four weeks to enhance the standing and stepping based on observation and electromyographic activity. After 278 sessions of epidural stimulation and physical training, both participants achieved over-ground walking, independent standing, and trunk stability.

## 7. Diagnosis and Treatment of Hand Function in Tetraplegia

**Figure 2:**
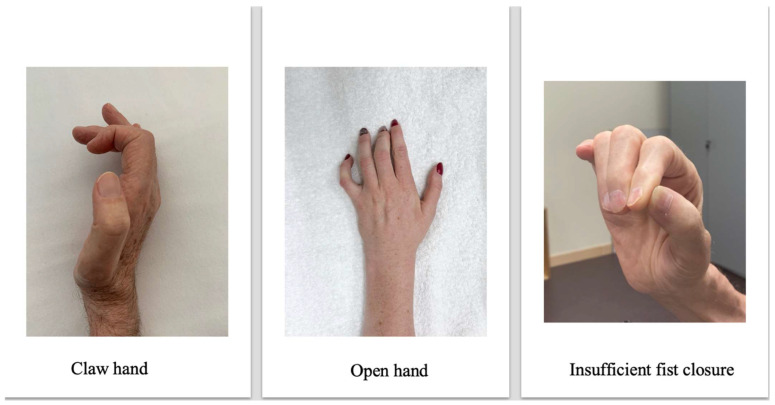
Hand shapes that hinder efficient grasping. Original source of the photo: International FES Centre^®^ Nottwil, Swiss Paraplegic Centre Nottwil, Switzerland.

**Figure 3:**
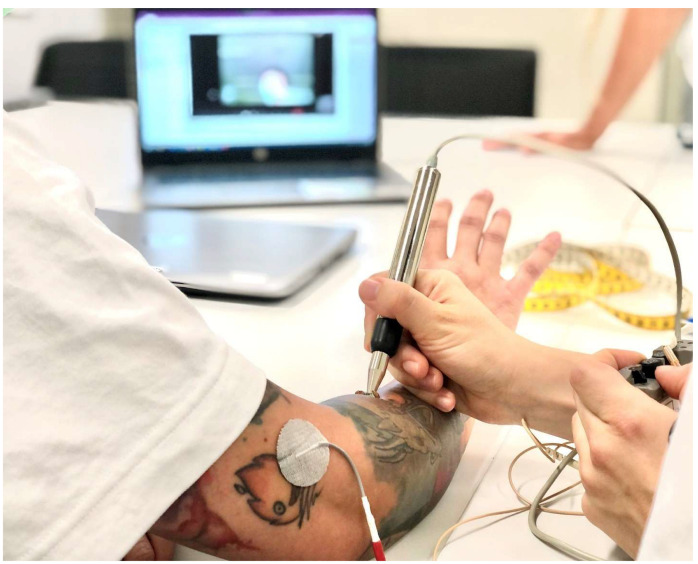
Motor point mapping of the extensor digitorum communis by applying stimulation with a pen electrode on the defined motor point. Original source of the photo: International FES Centre^®^ Nottwil, Swiss Paraplegic Centre Nottwil, Switzerland.

Regaining unrestricted use of the hand in gross and fine motor skills is one of the main health priorities after cervical SCI [71,72], with many regarding it as more important than walking [73,74]. The development of a tenodesis grip is the gold standard for people with tetraplegia in regaining hand function [75]. The tenodesis grip is based on a deliberate shortening of the long finger flexors causing flexion in the metacarpophalangeal and interphalangeal joints during active or passive dorsiflexion of the wrist. Thus, despite the absence of selective finger function, grasping and releasing objects can be achieved by opening the hand through volar flexion, taking an object in the open hand, and passively closing the hand through dorsiflexion in the wrist. Often, positioning, taping, and splinting in the first twelve weeks after injury are unreliable for the development of a tenodesis grip. Consequently, clawed hands, open hands, or insufficient fist closure may result (Figure 2). Neurophysiological factors such as damage to the lower motor neuron of key actuators for grasping can be the underlying mechanism [76,77]. To detect the underlying mechanism, electrical stimulation can be used as a diagnostic assessment. Stimulation of the defined motor points by means of motor point mapping [78] can be used with a pulse width of 300 µs, a frequency of 35 Hz, and a stimulation intensity of 20–60 mA to determine the integrity of the lower motor neuron (Figure 3). Thus, a standardized motor point mapping can reveal that a greater percentage of muscles on the dorsal aspect of the forearm show damage to either the lower or upper motor neuron. This finding may contrast with the flexors on the ventral aspect of the forearm, which may show significantly more partial damage to the lower motor neuron [8] and a less clear distribution between upper and lower motor neuron damage. Looking at the patterns of damage to the individual muscles of the forearm and hands in people with tetraplegia may be useful in predicting the development of different hand deformities. Testing within the first six to eight weeks after the onset of a SCI, motor point mapping can serve as a predictor for the development of voluntary muscle activity 24 weeks after injury [79]. 

**Figure 4:**
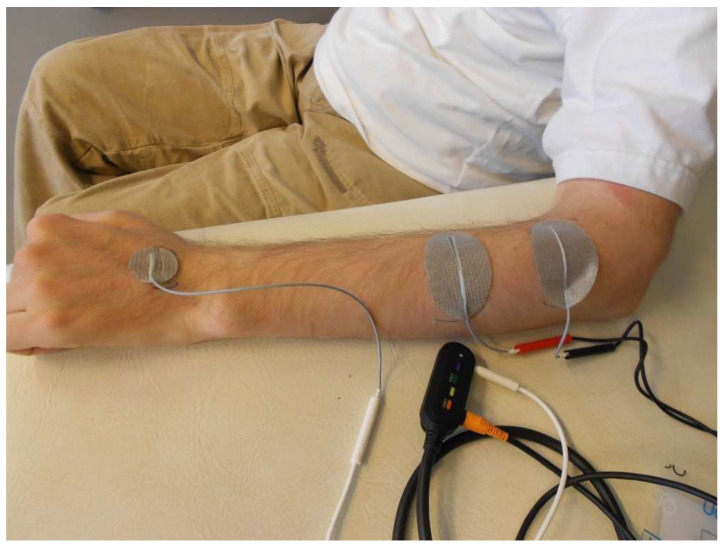
Example of an EMG-triggered stimulation of the hand and finger extensors. Original source of the photo: International FES Centre^®^ Nottwil, Swiss Paraplegic Centre Nottwil, Switzerland.

The implication for treatment is that functional and structural training with electrical stimulation can be started in a targeted and timely manner. So-called open hands usually present with lower motor neuron damage of the flexor digitorum profundus (FDP) and the extensor digitorum communis (EDC). In terms of muscle physiology, this type of damage presents a risk of denervation atrophy combined with the development of contractures in all finger joints. Timely stimulation of the denervated muscles with long pulse widths to maintain mobility and contractility of the muscles is recommended [80,81]. If the lower motor neurons are intact, synergistic balancing should be considered, and functional, task-oriented training, combined with EMG-triggered FES, should be started in a reasonable time. In the case of an intact lower motor neuron on the EDC resulting in increased reflex activity in this spinal segment due to upper motor neuron syndrome, and if the lower motor neuron of the FDP is damaged, a claw hand will likely develop. Often, taping the fingers in 90° flexion in the metacarpophalangeal joints stimulates muscle spindles, aggravating the condition and leading to claw hand [82,83]. Stimulation of the denervated FDP is recommended to maintain contractility and, secondarily, to avoid joint contractures, especially in the metacarpophalangeals. Denervation occurring on the EDC while the same lower motor neuron on the FDP as the antagonist is intact promotes the development of a tenodesis grip. FES of the wrist extensors with time-delayed stimulation of the finger flexors, triggered optimally via EMG, supports functional task-oriented training for learning to grasp with the tenodesis grip (Figure 4).

Electrical stimulation can be considered a diagnostic tool (motor point mapping) to detect eventual damage to the lower motor neuron. Identifying the type of damage at an early stage after the onset of SCI (six to eight weeks) serves as a predictor for the development of hand deformities 24 weeks after injury [79]. Prospectively, individualized treatment, as well as a targeted choice of appropriate stimulation parameters for the tetraplegic hand, can be applied in time.

## 8. Summary and Conclusions

As healthcare professionals, we have discussed evidence to help substantiate the importance of the use of electrical stimulation activities for individuals with SCI. These activities can play an important role during rehabilitation and as long-term activities to prevent secondary inactivity-associated conditions, such as cardiovascular and metabolic diseases. Additionally, electrical stimulation activities can help determine individual treatment strategies regarding hand function in individuals with cervical SCI. Further study is required to continue to fine-tune the dose-response relationships with rehabilitation and physical conditioning goals, and to discover new modalities to aid in the enhancement of the quality of life of people with SCI.
